# Off-Label-Use von Antibiotika in der Pferdemedizin – eine anonyme deutschlandweite Online-Umfrage

**DOI:** 10.1055/a-2585-3269

**Published:** 2025-06-13

**Authors:** Marie Tarillion, Robert Hertzsch, Angelika Richter

**Affiliations:** 1 Institut für Pharmakologie, Pharmazie und Toxikologie, Veterinärmedizinische Fakultät, Universität Leipzig; 2 Tierarzt Plus GmbH, Berlin

**Keywords:** Dosierungen, Prophylaxe, Tierarzneimittel, Therapieerfolg, Umwidmung, Zulassungsbedingungen, Doses, prophylaxis, veterinary medicinal products, therapeutic success, repurposing, summary of product characteristics

## Abstract

**Gegenstand und Ziel:**

Seit dem 28.01.2022 sind Tierarzneimittel gemäß Zulassungsbedingungen anzuwenden (VO (EU) 2019/6, Art. 106). Dies bringt weitere Einschränkungen in der Therapiefreiheit z. B. zu Dosisabweichungen mit sich, weil jede abweichende Anwendung von der Fachinformation ein Off-Label-Use darstellt. Zum Umfang des Off-Label-Uses von Antibiotika liegen bisher keine Daten in der Pferdemedizin vor, auf deren Basis die Umsetzbarkeit der neuen Reglementierungen in Deutschland eingeschätzt werden kann.

**Material und Methoden:**

Zur Erhebung der Daten zum Off-Label-Use von Antibiotika wurde daher eine deutschlandweite anonyme Online-Befragung als quantitative Querschnittsstudie durchgeführt, die bis zu 105 Fragen einschloss. Der Umfrage gingen Experteninterviews und Pilot-Umfragen voran.

**Ergebnisse:**

Insgesamt haben 111 Pferdetierärzte die Umfrage vollständig beantwortet. 88,3% (98/111) gaben an, Antibiotika off-label verwendet zu haben. Häufig umgewidmet wurden Enrofloxacin, Marbofloxacin, Breitspektrum-Cephalosporine, Doxycyclin und Metronidazol. Auch zugelassene Pferdearzneimittel mit Wirkstoffen wie Gentamicin, potenzierten Sulfonamiden, Benzylpenicillin-Procain und Oxytetrazyclin wurden off-label verwendet, oft hinsichtlich Indikation, Dosisintervall und Behandlungsdauer. Antibiotika wurden meist höher dosiert, selten prophylaktisch oder für nicht antibakterielle Zwecke genutzt. Beliebte Kombinationen waren Gentamicin mit Benzylpenicillin oder mit Amoxicillin. Humanmedizinische Antibiotika wurden selten eingesetzt.

**Schlussfolgerungen und klinische Relevanz:**

Wie diese Umfrage bestätigt, werden Antibiotika häufig nach Tierart und Indikation in der Pferdemedizin umgewidmet, was in zukünftigen weiteren Reglementierungen und der Überarbeitung der Positivliste berücksichtigt werden sollte. Besondere Bedingungen im Infektionsgeschehen rechtfertigen in vielen Fällen den Off-Label-Use auch von zugelassenen Präparaten für Pferde. Diese Umfrage gibt Hinweise darauf, dass manche Dosisrevisionen für jahrzehntelang eingesetzte Antibiotika, die als Arzneimittel für Pferde zugelassen sind, zu empfehlen sind. Da hierfür wenig finanzielle Anreize für pharmazeutische Unternehmen bestehen, könnte alternativ die Erstellung von Therapieleitfäden für bestimmte Indikationen bei Pferden zu mehr Sicherheit in der Pferdepraxis bezüglich der Begründungen eines Off-Label-Use beitragen.

## Abstract (en)

**Objective:**

Since 28.01.2022, veterinary medicinal products must be used in accordance with the conditions of authorization (Regulation (EU) 2019/6, Article 106). This entails further restrictions on therapeutic freedom, e. g., with regard to dose deviations. To date, there is no data available in equine medicine on the scope of off-label use as any use deviating from the expert information for antibiotics, on the basis of which the feasibility of implementing the new regulations in Germany can be assessed.

**Material and methods:**

To collect data on the off-label use of antibiotics, a Germany-wide anonymous online survey was therefore conducted as a quantitative cross-sectional study, which included up to 105 questions. The survey was preceded by expert interviews and pilot surveys.

**Results:**

A total of 111 equine veterinarians answered the survey in full. 88.3% (98/111) stated that they had used antibiotics off-label. Enrofloxacin, marbofloxacin, broad-spectrum cephalosporins, doxycycline and metronidazole were frequently repurposed. Approved drugs for horse such as gentamicin, potentiated sulfonamides, benzylpenicillin-procaine and oxytetracycline were also used off-label, often with regard to indication, dose interval and treatment duration. Antibiotics were usually used in higher doses, rarely prophylactically or for non-antibacterial purposes. Popular combinations were gentamicin with benzylpenicillin or with amoxicillin. Human medical antibiotics were rarely used.

**Conclusions and Clinical Relevance:**

As this survey confirms, there is a frequent need for repurposing of antibiotics according to animal species and indication in equine medicine, which should be taken into account in future further regulations and the revision of the positive list. In many cases, special conditions in the infection process justify the off-label use of approved preparations for horses. This survey indicates that some dose revisions are to be recommended for antibiotics that have been used for decades and are approved as medicinal products for horses. As there are few financial incentives for pharmaceutical companies to do so, the creation of therapy guidelines for certain indications in horses could alternatively contribute to greater certainty in equine practice with regard to the justification of off-label use.

## Einleitung

Mit der zum 28.01.2022 in Kraft getretenen Verordnung (EU) 2019/6 (Artikel 106 Absatz 1) sind Tierarzneimittel (TAM) gemäß ihrer Zulassungsbedingungen anzuwenden 1 . Off-Label-Use umfasst nicht nur die Abweichung von der Tierart und/oder der Indikation als sogenannte Umwidmung, sondern alle von der Kennzeichnung/Label/Fachinformation abweichenden Anwendungen, wie von der Applikationsart, Behandlungsdauer oder Dosierung, die auch maßgeblich von den pharmazeutischen Formulierungen eines TAM abhängen können 2 . Um unzumutbare Leiden abzuwenden, darf eine Umwidmung gemäß der über Artikel 112 (Equiden, die von der Schlachtung ausgenommen sind) bzw. Artikel 113 (Schlacht-Equiden) festgelegte Stufen erfolgen. Nur zugelassene TAM sind jedoch bezüglich der Angaben in der Kennzeichnung bzw. in der Fachinformation klinisch auf Wirksamkeit und Unbedenklichkeit geprüft. Insofern bestehen zum Off-Lable-Use berechtigte Bedenken bezüglich der Therapiesicherheit. Andererseits kann nicht nur die fehlende Verfügbarkeit zugelassener TAM, sondern insbesondere beim Einsatz von Antibiotika (AB) eine geringere Empfindlichkeit ursächlicher Bakterien oder Besonderheiten im Infektionsgeschehen (z. B. Verhältnisse am Infektionsherd) einen Off-Label-Use erforderlich machen, um Therapieerfolge zu erzielen 3 .

Über den tatsächlichen Off-Label-Use in der Tiermedizin ist für Deutschland in der Literatur wenig bekannt 4 . In einer nicht repräsentativen Umfrage in Deutschland, die sich ausschließlich auf die Umwidmung von Antiinfektiva bei Klein- und Nutztieren bezog, gaben 67% der 146 befragten Tierärzte an, systemisch wirksame AB abweichend von der Tierart und/oder der Indikation einzusetzen 5 . 15% der umgewidmeten systemisch wirksamen AB betrafen die der Gewinnung von Lebensmitteln dienenden, 4% die nicht der Gewinnung von Lebensmitteln dienenden Pferde. Andere Formen des Off-Label-Uses, wie Dosierungsabweichungen, wurden hierbei nicht abgefragt. Dies erscheint jedoch wichtig, denn Unterdosierungen und eine nicht angemessene Therapiedauer wären im Bestreben der Vermeidung von AB-Resistenzselektionen eher kontraproduktiv 6 . Tierärztliche Meldungen zu den AB-Verbrauchsmengen, die ab 2026/2027 auch den Einsatz bei Pferden unabhängig vom Equidenpass-Status einbeziehen (VO (EU) 2019/6, Artikel 57), werden nur begrenzte Einblicke in den Off-Label-Use in der Pferdemedizin gewähren. Hierüber sind zwar Informationen zu Umwidmungen in Bezug auf die Zieltierart zu erwarten, hingegen nicht zu Abweichungen von Dosierungen und Indikationen.

Daten zum Off-Label-Use bei einzelnen Tierarten können wichtige Indikatoren dafür sein, welche antibiotischen TAM im europaweiten Harmonisierungsverfahren der Fachinformationen (SPC-Harmonisierung nach Art. 69–72 der VO [EU] 2019/6) zu priorisieren sind. Zudem könnten daraus Hinweise abgeleitet werden, wo Forschungsbedarf für Dosisoptimierungen besteht. In einer Übersichtsarbeit zum Off-Label-Use von AB in der Tiermedizin rief die European Medicines Agency (EMA) 2017 dazu auf, Daten zum Off-Label-Use von AB, insbesondere für ausschließlich humanmedizinisch zugelassene AB, zu erarbeiten, um mögliche Therapielücken in der antibakteriellen Versorgung in der Tiermedizin aufzudecken 4 . Entgegen heutigen Anforderungen an die Zulassung wurden viele TAM mit antibakteriellen Wirkstoffen im Rahmen früherer Zulassungsverfahren nicht bezüglich Dosis und Wirksamkeit für einzelne pathogene Bakterien untersucht. Zudem ist durch einen jahrzehntelangen Einsatz vieler AB-Klassen eine Verminderung der Empfindlichkeit innerhalb von Bakterienpopulationen denkbar 3 . Es ist daher zu vermuten, dass die in den Zulassungsbedingungen antibiotischer TAM angeführten Dosierungen in der Praxis zur Behandlung zugelassener Indikationen teilweise nicht (mehr) wirksam sind. Tierärztliche Meldungen zur mangelnden Wirksamkeit bei bestimmungsgemäßer Anwendung sind jedoch rar und beschreiben i.d.R. Einzelfälle mit wenig Aussagekraft, so dass im Rahmen des Pharmakovigilanz-Systems keine Überprüfungen z. B. von Dosierungen veranlasst werden können 7 .

Aus den oben genannten Gründen lag der Fokus dieser Umfrage auf dem Off-Label-Use von antibakteriell wirksamen TAM, die für Pferde zugelassen sind, wie Abweichungen zu Dosierungsvorgaben der Fachinformation. Außerdem wurde die Umwidmung von TAM, die für andere Tierarten zugelassen sind, sowie von antibakteriell wirksamen Humanarzneimitteln (HM) abgefragt, um Informationen zum möglichen Bedarf an zugelassenen Pferdearzneimitteln zu erhalten. Erfasst wurde zudem die Häufigkeit des Einsatzes von AB-Kombinationen, die nicht als Kombinationspräparate (sog. fixe Kombinationen) zugelassen sind, weil solche nicht fixen Kombinationen hinsichtlich der Selektion von Resistenzen in der Kritik stehen 6 . Auch der prophylaktische Einsatz sowie Anwendung zu nicht antibakteriellen Zwecken von AB wurden in die Umfrage eingeschlossen, um hierzu erstmals Informationen zum Umfang und Begründungen zu erhalten. Die antibakterielle Prophylaxe – d. h. die Anwendung eines AB bevor klinische Anzeichen einer Erkrankung auftreten, soll auf einzelne Tiere oder kleine Gruppen von Tieren im Falle eines hohen Infektionsrisikos beschränkt werden (VO [EU] 2019/6, Art. 107) – kann in Einzelfällen somit berechtigt sein. Die finale Abfrage erfolgte von März 2023 bis Januar 2024, d. h. im Zeitraum nach Inkrafttreten des neuen TAM-Rechts, um eine Basis zur Einschätzung möglicher Probleme durch das Zulassungsprimat sowie weiterer rechtlicher Vorgaben, die aktuell bezüglich Umwidmungsverboten beraten werden, zu schaffen. Diesbezüglich wurden die praktizierenden Pferdemediziner befragt, ob sie durch die Vorgaben der VO (EU) 2019/6 eine Gefährdung von Therapieerfolgen sehen.

## Material und Methoden

Zur Erhebung der Daten zum Off-Label-Use in der Pferdemedizin wurde eine anonyme, deutschlandweite Online-Befragung durchgeführt. Es handelt sich dabei um eine quantitative Querschnittsstudie, die eine Momentaufnahme darstellt 8 9 .

### Vorarbeiten zur Erstellung der Umfrage

Zunächst wurde eine Präparate-Wirkstoff-Tabelle mit den Anwendungsvorgaben in Excel (Microsoft Excel LTSC MSO [16.0.14332.20529] 64-Bit) angefertigt, die die Grundlage für die weiterführenden Schritte darstellte ( **Zusatz-Material 1** ). Sie beinhaltete alle 27 systemisch anzuwendenden AB-haltigen TAM mit insgesamt 10 verschiedenen Wirkstoffen, die für Pferde zu dem Zeitpunkt in Deutschland zugelassen und auf dem Markt verfügbar waren (Stand: 13.07.2022, Recherche über VETIDATA). In Tab. 1 unter „Detailfragen – antibiotische Wirkstoffe“ wurde Amoxicillin aufgrund unterschiedlicher Formulierungen (Injektionslösungen mit und ohne verzögerter Wirkstofffreisetzung) doppelt berücksichtigt. Lokal anzuwendende AB wurden nicht berücksichtigt. Für die systemisch anzuwendenden Präparate wurden dabei die in der Fachinformation genannten Zulassungsbedingen erfasst: Applikationsart, Behandlungsdauer, Dosis, Indikation und Verabreichungsfrequenz. Dabei wurde die gesamte Spanne der zugelassenen Anwendungen durch Minimal- und Maximal-Werte berücksichtigt, z. B. ein zugelassener Dosierungsbereich von 3–10 mg/kg je Einzeldosis. In der späteren Umfrage wurden alle zugelassenen Anwendungsvorgaben zu einem „wie zugelassen: (…)“ zusammengefasst und in gleichmäßigen Abstufungen davon abweichende Antwortoptionen darüber und darunter angeordnet, z. B. für Oxytetrazyclin: „< 1,5 mg/kg“, „1,5–2,9 mg/kg“, „wie zugelassen: 3–10 mg/kg“, „11–15 mg/kg“, „16–20 mg/kg“ und „> 20 mg/kg“.

**Table:** **Tab. 1** Übersicht über die Anordnung der Fragenblöcke und der Anzahl der Fragen. **Table 1** Overview of the arrangement of the question blocks and the number of questions.

Fragenblock	Fagenanzahl	
	Maximal	Minimal
Demographische Fragen	8	8
Allgemeine Fragen	11	4
Prophylaxe	3	1
Einsatz für nicht antibakterielle Zwecke	4	1
Nicht als Fertigarzneimittel zugelassene AB-Kombinationen	6	2
Humanmedizinische AB	4	2
Therapieerfolg	3	3
Detailfragen – antibiotische Wirkstoffe	11* x 6	–
Insgesamt	39+66	21

*Hierbei handelte es sich um 10 verschiedene Wirkstoffe; für einen Wirkstoff wurden 2 verschiedene Formulierungen separat abgefragt. *These included 10 compounds, considering for one compound 2 different formulations.

Im Zuge der Entwicklung der finalen Umfrage wurden vorab 9 Experteninterviews zur Fragenfindung zum Thema Off-Label-Use von AB durchgeführt. Sie dienten auch als Vorab-Test 8 10 . Aus den daraus gewonnenen Erkenntnissen und Themenschwerpunkten wurde im Anschluss im Online-Umfrage-Tool Lime Survey die erste Umfrage-Version erstellt. Die quantitative Pilot-Umfrage richtete sich von Dezember 2022 bis Februar 2023 an insgesamt 25 Tierärzte mit dem Behandlungsschwerpunkt Pferde. Gegenstand der Pilot-Umfrage waren neben den eigentlichen thematischen Fragen auch sog. Metafragen ( **Zusatz-Material 2** ; https://www.cmaj.ca/content/suppl/2008/07/24/179.3.245.DC1 ) 8 . Diese überprüfen den Inhalt der Umfrage auf die Relevanz der Fragen und in Hinblick auf Angemessenheit, Klarheit und Verständlichkeit („ *clinical sensibility testing* “) 8 . Im Zuge der Überarbeitung der Umfrage anhand der Ergebnisse aus der Pilot-Umfrage wurden Antwortoptionen ergänzt, Fragen umformuliert bzw. konkretisiert, die Anordnung der Fragen und Fragenblöcke teilweise geändert, redundante Fragen entfernt und Hilfetexte bzw. Erklärungen ergänzt.

### Durchführung der Umfrage

#### Fragenblöcke

Tab. 1 zeigt die Anordnung der Fragenblöcke mit der Anzahl der Fragen. Der Fragebogen kann online eingesehen werden ( **Zusatz-Material 2** ).

**Abb. 1:**
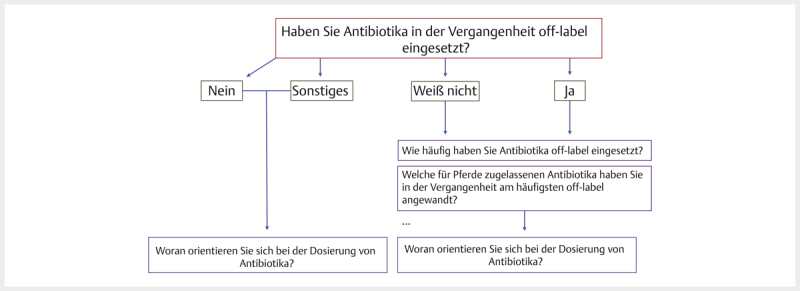
Entscheidungsbaum; die Abbildung zeigt beispielhaft die Verknüpfung der Fragen abhängig von den gewählten Antwortoptionen vorangegangener Fragen. Das eingestellte Rückwärtsnavigieren ermöglichte den Teilnehmern ein nachträgliches Ändern der Antworten. So konnten „unsichere“ Teilnehmer die Antwortoption „weiß nicht“ wählen und so trotzdem zu den Anschlussfragen gelangen. Durch die Anzeige der Antwortoptionen der Anschlussfrage konnten Teilnehmer Gewissheit darüber erlangen, ob sie z. B. Antibiotika off-label angewandt haben oder nicht. Dadurch hatten Teilnehmer die Chance, doch noch den „richtigen Weg“ in der Umfrage einzuschlagen. Quelle: M. Tarillion. **Fig. 1** Decision tree; the figure shows an example of how the questions are linked depending on the answer options selected for previous questions. The backward navigation setting allowed participants to change their answers retrospectively. This allowed “insecure” participants to select the “donʼt know” answer option and still get to the follow-up questions. By displaying the answer options of the follow-up question, participants could gain certainty as to whether or not they had used antibiotics off-label, for example. This gave participants the chance to take the “right path” in the survey after all. Source: M. Tarillion.

Am Anfang des Fragebogens wurde zunächst gefragt, ob die Teilnehmer AB überhaupt innerhalb der letzten 2 Jahre off-label eingesetzt haben. Je nach Antwort nahm der Teilnehmer automatisch einen entsprechenden „Weg“ im Verlauf der Umfrage ( Abb. 1 ). Die maximale Anzahl der Fragen wurde nur erreicht, wenn alle bedingten Fragen durch die Auswahl einer bestimmten Antwortoption „aktiviert“ wurden ( Abb. 1 ). In der dritten Spalte der Tab. 1 ist die Minimalanzahl der Fragen zu sehen, wenn keine der bedingten Fragen „aktiviert“ wurde. In der vorletzten Zeile wird die Anzahl der Detailfragen aufgezeigt.

### Fragenformate

Die Umfrage enthielt überwiegend Multiple-Choice-(MC-) (25 Fragen) und Single-Choice-(SC-)Fragen (71 Fragen). Außerdem wurden einzelne Fragen des Typs Ja-Nein-Frage (7 Fragen), Fünf-Punkt-Auswahl (1 Frage) und Reihenfolge (1 Frage) verwendet. Bei dem Fragentyp „Reihenfolge“ wurden die Befragten aufgefordert, die Antwortoptionen in eine bestimmte Reihenfolge zu bringen.

### Beispiele für Auswahlmöglichkeiten

**Abb. 2:**
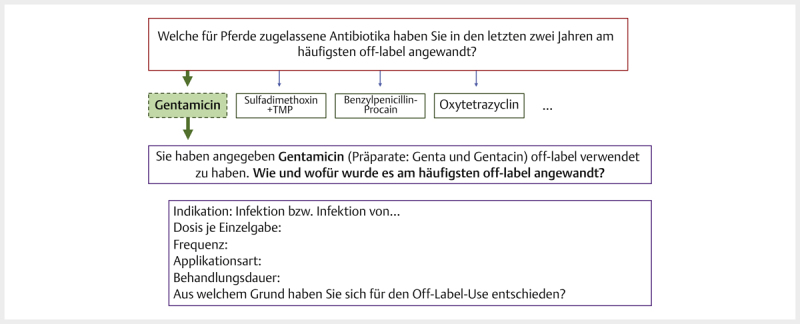
Entscheidungsbaum für Detailfragen zu Wirkstoffen: Bei Auswahl der Antwortoption „Gentamicin“ (gestrichelter grüner Kasten), wurde der Teilnehmer zu detaillierten Fragen zur Dosierung und Anwendung von „Gentamicin“ weitergeleitet (untersten beiden Kästen mit violettem Rahmen). Quelle: M. Tarillion. **Fig. 2** Decision tree concerning detailed questions on active ingredients: The choice of the option “Gentamicin” (dashed green box) led to detailed questions on the dosage and use of gentamicin later in the survey (bottom two boxes with purple frame). Source: M. Tarillion.

Nur die Wirkstoffe, die man bei einer vorherigen Frage ausgewählt hatte, wurden dort im Detail behandelt, so dass deren Anzeige von der Vorauswahl der Wirkstoffe in einer vorhergegangenen Frage abhing ( Abb. 2 ). Wählte der Teilnehmer dort „Ja“ aus, gelangt er an einem bestimmten Punkt der Umfrage zu der Frage, die schematisch in Abb. 2 dargestellt ist. Wird bei dieser z. B. der Wirkstoff „Gentamicin“ (im gestrichelten Kasten) ausgewählt, wird der befragten Person zur gegebenen Zeit die Detailfrage für „Gentamicin“ (violett umrandete Fragen) angezeigt ( Abb. 2 ).

### Rekrutierung von Teilnehmern/Veröffentlichung der Umfrage

Die Verbreitung der Final-Umfrage erfolgte über eine Anzeige im Deutschen Tierärzteblatt, über Rundmails der Tierärztekammern, über verschiedene tiermedizinische Gesellschaften (z. B. Gesellschaft für Pferdemedizin, GPM), auf Tierärztekongressen und über das Kontaktieren einzelner Pferdetierärzte in Deutschland und über weitere Kanäle (z. B. BVL-Newsletter). Einzelne Pferdetierärzte wurden durch eine Google-Maps-Suche zufällig ausgewählt.

## Prüfung auf Repräsentativität und Auswertung

### Prüfung auf Repräsentativität

Der Umfragezeitraum erstreckte sich von Ende März 2023 bis Januar 2024. Zur Einschätzung der repräsentativen Anzahl diente als Grundlage die Gesamtheit aller für die Beantwortung der Umfrage in Betracht zu ziehenden Personen, die über die Statistik zur Tierärzteschaft in der Bundesrepublik Deutschland für das Jahr 2022 ermittelt wurde 11 . So wurden die Zahlen der Statistik aus der Kategorie „Praktisch Tätige nach Tierarten“ aus „Pferde“ entnommen. Pferdepraktiker aus Kategorien mehrerer Tierarten, z. B. „Kleintier und Pferde“, wurden nicht berücksichtigt. Für die Pferde ergab sich eine Grundgesamtheit von 1030 Tierärzten (Niedergelassene: 866, Assistenten: 163, Praxisvertreter: 1). Bei Berechnung der Stichprobengröße mit Hilfe des Stichprobenrechners Epitool ( https://epitools.ausvet.com.au/samplesize ) läge bei einem Konfidenzniveau von 95%, einer Fehlerspanne von 5% die Zielgröße zwar bei 246, jedoch konnten mit insgesamt 111 Teilnehmern rund 10% aller in Deutschland tätigen Pferdepraktiker bezüglich des Themas Off-Label-Use von AB in der Pferdemedizin befragt werden.

### Auswertung der Umfrage

Die Auswertung der Umfrage erfolgte in der Programmiersprache R in der Oberfläche RStudio. Bei der rein deskriptiven Auswertung wurden Häufigkeitstabellen erstellt. Die Daten wurden dazu als csv-Datei aus Lime Survey exportiert und zur Auswertung in RStudio importiert. Die Darstellung der Graphen erfolgte mit Hilfe von Microsoft Excel (Microsoft Excel LTSC MSO (16.0.14332.20734) 64-Bit).

## Ergebnisse

Insgesamt wurde die Final-Umfrage von 254 Tierärzten beantwortet, was bezogen auf die Gesamtheit der 1030 in Deutschland praktizierenden Pferdemedizinern 11 einer Antwortrate von 24,7% entspricht. Allerdings beantworteten nur 111 (43,7% von 254) die Umfrage vollständig, 143 (56,3%, 143/254) brachen den Fragebogen vorzeitig ab. Der Abbruch des Fragebogens erfolgte überwiegend schon bei den demographischen Fragen. Im Mittel wurde der Fragebogen zwischen den Allgemeinen Fragen (zweiter Fragenblock, Tab. 1 ) und den Fragen nach dem prophylaktischen Einsatz von AB (dritter Fragenblock, Tab. 1 ) abgebrochen. Bei den verbleibenden 111 Teilnehmern, deren Antworten in die Auswertung eingingen, blieben nur einzelne Fragen unbeantwortet, wie nachfolgend angegeben. Somit ist die Umfrage bezüglich der Repräsentativität limitiert. Von den insgesamt 111 Teilnehmern gab der überwiegende Teil der Pferdepraktiker an, selbstständig tätig zu sein (53,1%, 59/111), im Bereich Allgemeinmedizin zu arbeiten (64%, 71/111) und schon über 20 Jahre Berufserfahrung zu haben (43,2%, 48/111). Die meisten Teilnehmer praktizierten in Niedersachsen (21/111), Nordrhein-Westfalen (20/111) und Bayern (19/111).

Die mittlere Antwortzeit betrug 18 und im Median 13 Minuten. Die Umfragedaten wurden auf sog. „Raser“, d. h. Teilnehmer, die durch schnelles Durchklicken der Umfrage eine unrealistisch kurze Beantwortungszeit (durch Probeläufe auf weniger als 2 Minuten definiert) aufweisen und auf sog. „Straightliner“, d. h. Teilnehmer, die Antworten immer an gleicher Position, z. B. immer die erste Antwortoption, wählen, überprüft 12 . Im Zuge der Auswertung konnten weder „Raser“ noch „Straightliner“ ausfindig gemacht werden.

### Angaben zum Off-Label-Use

**Abb. 3:**
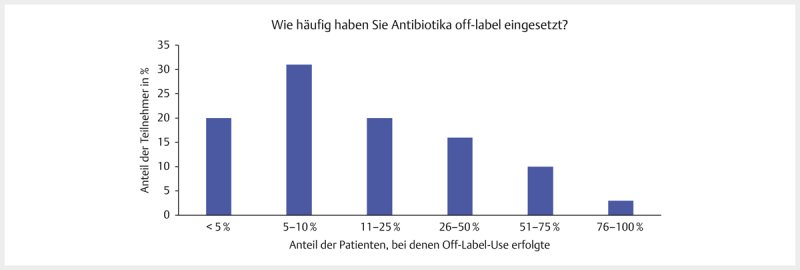
„Wie häufig haben Sie Antibiotika off-label eingesetzt?“ (bezogen auf Anteil der Patienten); Prozentuale Verteilung bezogen auf die Teilnehmerzahl, n=100, 98 sowie 2 mit der Antwort „Weiß nicht“, SC-Frage. Quelle: M. Tarillion. **Fig. 3** “How often have you used antibiotics off-label?”; Percentage distribution in relation to the number of participants, n=100, 98 and 2 with the answer “Donʼt know”, SC question. Source: M. Tarillion.

88,3% (98/111) der befragten Tierärzte gaben an, AB off-label einzusetzen. Zur Häufigkeit antworteten nur 100 von 111 Teilnehmer. 31% (31/100) gaben an, AB bei 5–10% ihrer Patienten off-label eingesetzt zu haben. 49 Tierärzte machten häufigeren Gebrauch vom Off-Lable-Use, 20 hingegen seltener ( Abb. 3 ). Die 3 häufigsten Informationsquellen für den Off-lable-Use, wie zu Dosierungen von AB, waren Fachbücher (82%), Packungsbeilage (76,6%) und Leitlinien (34,2%). Die Gewichtsbestimmung der Pferde als Grundlage für die AB-Dosierung erfolgte zu fast gleichen Teilen durch Schätzungen (36,9%, 41/111) oder durch Wiegen (36%, 40/111). Achtzehn Tierärzte verwendeten ein spezielles Maßband zur Gewichtsbestimmung und 14 den Body Condition Score.

Die häufigsten Gründe für einen Off-Label-Use im Allgemeinen, die in einer separaten Frage mit 10 Antwortoptionen abgefragt wurden, waren in absteigender Reihenfolge „Dosis“ (605 Punkte), „Packungsgröße“ (521 Punkte), „Verabreichungsfrequenz“ (467 Punkte) und „Indikation“ (465 Punkte). Weitere Gründe waren „Therapiedauer“ (451 Punkte), „Applikationsart“ (429 Punkte), „Verfügbarkeit“ (413 Punkte), „Therapienotstand“ (352 Punkte), „Darreichungsform“ (316 Punkte) und an letzter Stelle „Kosten“ (242 Punkte). Die Punkte ergeben sich aus dem Fragentyp „Reihenfolge“, bei dem die Teilnehmer die Antwortoptionen in eine nach Wichtigkeit absteigende Reihenfolge bringen sollten. Die Auswertung erfolgte gewichtet, wobei die Positionen verschiedene Faktoren erhielten. Zum Beispiel wurden Antwortoptionen an Position Eins mit dem Faktor Zehn gewichtet und Antwortoptionen an unterster Position mit Faktor Eins.

### Off-Label-Use von antibakteriell wirksamen TAM, die für Pferde zugelassen sind

In Tab. 2 sind Angaben zu AB, die für Pferde als zugelassene TAM in Deutschland verfügbar waren, zusammengefasst. 100 Teilnehmer beantworteten die Frage, welche Wirkstoffe am häufigsten off-label eingesetzt wurden. Der häufigste Off-Label-Use erfolgte bei Präparaten mit der fixen Kombination aus einem Sulfonamid und Trimethoprim (TMP) (81%, 81/100), bei Präparaten mit dem Aminoglykosid Gentamicin (42%, 42/100), gefolgt vom Depot-Penicillin Benzylpenicillin-Procain (37%, 37/100) sowie Oxytetrazyclin (33%, 33/100). Unter den beiden systemisch wirksamen zugelassenen Amoxicillin-haltigen Präparaten wurde sowohl das Präparat zur einmaligen Initialbehandlung und die beiden anderen Präparate zur Langzeitbehandlung von 14% der 100 Tierärzte off-label eingesetzt.

**Table:** **Tab. 2** Anzahl der Teilnehmer, die die Frage zum Off-Label-Use der für Pferde zugelassenen AB beantwortet haben (n=100) mit der Anzahl der zugelassenen Präparate (n). Die Art des Off-Label-Uses mit der Anzahl der Teilnehmer, die die jeweilige Art angaben, betraf die Indikation im Sinne einer Umwidmung sowie die Applikationsart, die Erhöhung der Dosis (↑) bzw. der Frequenz der Verabreichung (Verkürzung des Dosierungsintervalls), in Einzelfällen (selten n≤2) auch Verminderung der Dosis (↓); keine Angabe (k.A.). **Table 2** Number of participants that answered the question on off-label use of antibiotics approved for horses (n = 100) with the number of approved preparations (n). The type of off-label use with the number of participants chosing a specific type of off-label use (n). Off-label use concerned the indication in the sense of a reclassification as well as the type of application, higher dose (↑) or frequency of administration (shortening of the dosing interval) or in few cases lower dose (↓ n ≤ 2). no reply (k.A).

Wirkstoff in den für Pferde zugelassenen TAM (n)	Anzahl der Teilnehmer (n)	Art des Off-Label-Uses (n)
Potenzierte Sulfonamide (13)	80	Indikation (31) Frequenz (↑=23) Dosis (↑=17) Dauer (↑=18) Applikationsart (9)
Gentamicin (2)	42	Indikation (40) Dosis (↑=4, selten↓=1)
Benzylpenicillin-Procain (5)	37	Indikation (27) Dauer (↑=16) Dosis (↑=10, selten↓=1)
Oxytetrazyclin (1)	33	Indikation (28) Dauer (↑=12) Dosis (↑=8)
Amoxicillin, Präparat 100 mg/ml (1)	14	Indikation (8) Frequenz (↑=11) Dauer (↑=10) Dosis (↑=3, selten↓=1)
Amoxicillin, Präparat 200 mg/ml (1)	14	Indikation (12) Dauer (↑=9) Dosis (↑=3)
Ampicillin (1)	1	Indikation (1)
Sulfadimidin (3)	1	k.A.

Wie in Tab. 2 genauer zusammenfasst, unterschieden sich die Art des Off-Label-Uses und die Häufigkeiten für die Gründe bei den einzelnen Wirkstoffen. Vordergründig waren demnach: „Sofortige Behandlung vor bakteriologischem Untersuchungsergebnis notwendig“, „kein TAM für die Indikation zugelassen“, „Mischinfektionen“ und „keine ausreichende Wirkung aufgrund nicht optimaler Dosierung laut Zulassung“.

Als Gründe für die Umwidmung der Indikation wurden vor allem Abszesse (58/100), Infektionen des Respirationstrakts (51/100), Infektionen des Reproduktionstrakts (36/100), Septikämie (36/100) und Infektionen von Gelenken und Knochen (33/100) angegeben. Nachfolgend werden die Art des Off-label-Uses sowie die Begründungen für die besonders häufig off-label eingesetzten AB genauer beschrieben.

Die **potenzierten Sulfonamide** wurden am häufigsten off-label angewendet. Sie sind für Pferde mit einer Dosis von 15–30 mg/kg bezogen auf die Gesamtwirkstoffmenge für 3 bis 7 Tage zur oralen oder parenteralen Applikation zugelassen. Die zugelassenen Präparate decken die Indikationen Infektionen von Gelenken und Knochen, Haut und Hautanhangsorganen, Infektionen des Respirations-, Gastrointestinal- und Urogenitaltrakts ab. Sulfonamide in Kombination mit TMP wurden nach Indikation meistens zur Behandlung von „Abszessen, Phlegmonen und Wunden“ umgewidmet (40,5%, 15/37). Häufiger bestand der Off-Label-Use bei der Frequenz der Verabreichung. So gab der Großteil der Tierärzte an, potenzierte Sulfonamide zweimal täglich in der empfohlenen Tagesdosis statt wie zugelassen einmal täglich eingesetzt zu haben. Bei der Behandlungsdauer überwog zwar die zugelassene Anwendung von 3 bis 7 Tagen, jedoch gaben auch 41,3% (33/80) an, Sulfonamide+TMP länger als zugelassen angewandt zu haben. Als Grund für den Off-Label-Use wurde an erster Stelle „keine ausreichende Wirkung aufgrund nicht optimaler Dosierung laut Zulassung“ mit 37,5% (30/80) genannt, gefolgt von „kein TAM zugelassen“ mit 23,8% (19/80).

**Abb. 4:**
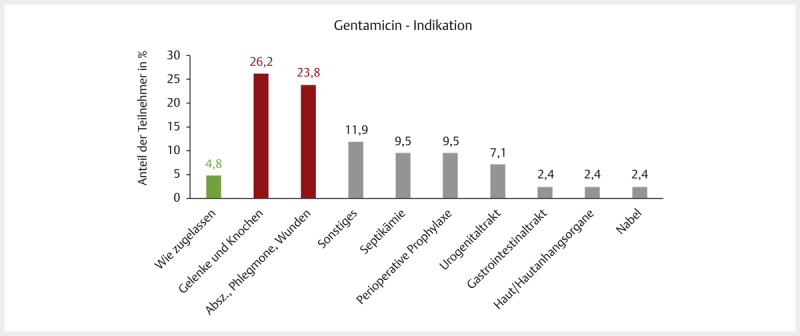
Detailfrage zur Off-label-Indikation von Gentamicin („wofür wurde es hauptsächlich angewandt?“). Prozentuale Verteilung der Indikationen bezogen auf die Teilnehmerzahl, n=42. In Grün die zugelassene Indikation bei Infektionen des Respirationstrakts und rechts daneben in Rot die beiden meist gewählten Indikationen, die von der zugelassenen abweichen. SC-Frage. Quelle: M. Tarillion. **Fig. 4** Detailed question on the off-label indication of gentamicin (“for which indication was this mainly used off-label? “), percentage distribution of indications in relation to the number of participants, n=42. In green the approved indication for respiratory tract infections and on the right in red the two most frequently selected indications that deviate from the approved indication. SC question. Source: M. Tarillion.

Die Detailfragen zu **Gentamicin** beantworteten 42 Teilnehmer. Zugelassen waren für Pferde 2 Präparate zur intravenösen Applikation ausschließlich zur Behandlung von Infektionen der unteren Atemwege in einer Dosis von einmal täglich 6,6 mg/kg für 3 bis 5 Tage. Als Grund für den Off-label-Use der Gentamicin-haltigen Präparate wurde hier mit 35,7% (15/42) am häufigsten „Sofortige Behandlung vor bakteriologischem Untersuchungsergebnis notwendig“ (meist verbunden mit der Kombination plus Penicillin) gewählt, gefolgt von „kein für das jeweilige Anwendungsgebiet TAM zugelassen“ mit 33,3% (14/42). Die Off-Label-Indikation für Gentamicin sind in Abb. 4 dargestellt.

Als der am dritthäufigsten off-label eingesetzte antibakterielle Wirkstoff, der für Pferde zugelassen ist, stellte sich **Benzylpenicillin-Procain** heraus, für das zum Zeitpunkt der Umfrageerstellung 5 systemisch wirksame Präparate zugelassen waren, die die Indikationen bei Infektionen von Gelenken und Knochen, Urogenital-, Respirationstrakt, Haut und Hautanhangsorgane und bei Septikämie mit einer zugelassenen Dosierung von 15–21 mg/kg bzw. 15000–20000 I.E. je Einzelgabe abdeckten. Dieser Fragenblock wurde von 37 der 111 befragten Tierärzte beantwortet. Der Off-Label-Use überwog bei der Indikation und der Behandlungsdauer. Mit 43,2% (16/37) wurde Benzylpenicillin-Procain von den meisten länger als 3 bis 4 Tage angewendet, was jedoch nur für ein zugelassenes Präparat den Zulassungsbestimmungen entspricht. Begründet wurde der Off-Label-Use von den meisten dadurch, dass die Notwendigkeit einer sofortigen Behandlung vor dem bakteriologischen Untersuchungsergebnis bestand (43,2%, 16/37). Mit jeweils 27% (10/37) folgten die Antwortoptionen „Mischinfektionen“ und „keine ausreichende Wirkung aufgrund nicht optimaler Dosierung laut Zulassung“, was mit der abweichenden Behandlungsdauer in Verbindung stand. Außerdem zeigen die Antworten zur Dosierung von Benzylpenicillin-Procain, dass die Teilnehmer neben der überwiegenden zugelassenen Dosierung von „15–21 mg/kg bzw. 15000–20000 I.E.“ (62,2%, 23/37) auch höhere Dosierungen als zugelassen ausgewählt haben: „21,1–31,5 mg/kg bzw. 20001–30000 I.E.“ (16,2%, 6/37) und „31,6–42 mg/kg bzw. 30001–40000 I.E.“ (10,8%, 4/37). Aufgrund der abweichenden Indikation wäre als Ursache für den Off-Label-Use eher die Antwortoption „kein TAM zugelassen“ zu erwarten gewesen. Dies spiegelt sich jedoch nicht in den Ergebnissen wider.

**Oxytetrazyclin** wurde von 33,3% der 100 Tierärzte, die diese Frage beantworteten, off-label eingesetzt. Zum Zeitpunkt der Umfrageerstellung existierte ein einziges systemisch wirksames für Pferde zugelassenes Präparat zur intramuskulären und intravenösen Applikation. Dieses war mit 3–10 mg/kg zur einmal täglichen Behandlung gegen Oxytetrazyclin-empfindliche Erreger zugelassen. Die 3 meistgenannten Gründe, die zum Off-Label-Use von Oxytetrazyclin geführt haben, waren „kein TAM für die Indikation zugelassen“ (51,5%, 17/33), „Sonstiges“ (24,2%, 8/33) und „keine ausreichende Wirkung aufgrund nicht optimaler Dosierung laut Zulassung“ (24,2%, 8/33). Die Antwortoptionen „Sonstiges“ (30,3%, 10/33) und „Infektionen von Gelenken und Knochen“ (24,2%, 8/33) waren die häufigsten beiden Antworten. Unter „Sonstiges“ fanden sich 10 Freitextkommentare: „zur Sehnenerweichung bei Sehnenstelzfuß (Fohlen)“ (8/10), „Verdacht auf Leptospirose“ (1/10) und „lokal am Auge“ (1/10). Andere Kommentare waren z. B.: „One-Shot-Antibiose prä-OP“, „Anaplasmose längere Therapiedauer“. Für die Behandlungsdauer gaben die meisten an, Oxytetrazyclin länger als zugelassen angewendet zu haben (38,7%, 12/33).

### Umwidmung von antibakteriell wirksamen TAM, die für Pferde nicht zugelassen sind

Unter den Wirkstoffen, für die es keine zugelassenen TAM für Equiden, sondern nur für andere Tierarten gibt ( Tab. 3 ), wurden die Fluorchinolone Enrofloxacin und Marbofloxacin sowie die Breitspektrum-Cephalosporine Cefquinom (4. Generation) und Ceftiofur (3. Generation) von vielen Teilnehmern umgewidmet, zudem auch Doxycyclin und Metronidazol.

**Table:** **Tab. 3** Antworten zur MC-Frage: „Welche tiermedizinisch zugelassenen Antibiotika, die nicht für Pferde zugelassen sind, haben Sie in den letzten 2 Jahren angewandt?“, absolute Häufigkeit, n=100. **Table 3** Answers to the MC question: “Which veterinary antibiotics that are not approved for horses have you used in the last 2 years?“, absolute frequency, n=100.

Wirkstoffe	Häufigkeit absolut (bei 100 Teilnehmern)
Enrofloxacin	64
Doxycyclin	52
Marbofloxacin	43
Cefquinom	37
Metronidazol	35
Ceftiofur	32
Tetracyclin	20
Tulathromycin	15
Chloramphenicol	13

Wenige Tierärzte (1–9) setzten in abfallender Reihenfolge auch ein: Neomycin, Dihydrostreptomycin, Cefalexin, Chlortetrazyklin, Florfenicol, Orbifloxacin, Furazolidon, Fusidinsäure, Lincomycin, Penethamat, Bacitracin, Cefoperazon, Colistin, Danofloxacin, Dimetridazol, Nafcillin, Oxacillin, Pradofloxacin, Ronidazol, Spiramycin umgewidmet, zudem auch Polymyxin B, das jedoch nur zur lokalen Anwendung als TAM zugelassen ist. Andere tiermedizinisch zugelassene AB wurden von den Teilnehmern nicht umgewidmet. Zur Vermeidung eines zu großen Fragenumfangs wurden keine Fragen zu Anteilen der Patienten gestellt. Gründe ergaben sich für Umwidmungen aus der mangelnden Verfügbarkeit zugelassener TAM für die Indikation.

### Umwidmung von antibakteriell wirksamen Humanarzneimitteln

108 von 111 Teilnehmern beantworteten die Frage nach der Relevanz von humanmedizinischen AB in der Pferdemedizin. Von diesen gaben 50% an, humanmedizinische AB schon umgewidmet zu haben. Dies erfolgte bei 40,7% (22/54) bei 1–5% der Patienten und bei 25,9% (14/54) bei weniger als 1% der Patienten.

**Abb. 6:**
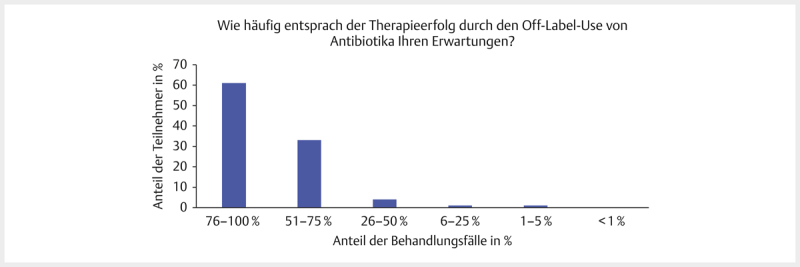
„Wie häufig entsprach der Therapieerfolg durch den Off-Label-Use von Antibiotika Ihren Erwartungen (in Prozent der Behandlungsfälle)?“ Teilnehmerzahl, n=100, SC-Frage. Quelle: M. Tarillion. **Fig. 6** “How often did the therapeutic success of off-label use of antibiotics meet your expectations (% of cases)?” Number of participants, n=100, SC question. Source: M. Tarillion.

Hierzu wurden keine Wirkstoffe abgefragt, weil die Liste der Antwortoptionen aufgrund vieler humanmedizinisch eingesetzter Antiinfektiva zu lang gewesen wäre. Stattdessen wurde die Frage gestellt, ob sich durch die Durchführungsverordnung (EU) 2022/1255, nach der bestimmte AB ausschließlich der Humanmedizin vorbehalten sind, Therapieprobleme bei Pferden ergeben. Laut Umfrage stellt das Einsatzverbot dieser Wirkstoffe für die Mehrzahl der Teilnehmer (76,6%; 82/107) kein therapeutisches Problem dar.

### Angaben zum prophylaktischen und nicht antibakteriellen Einsatz

Zum Zeitpunkt der Umfrageerstellung gab es für Pferde kein zur Prophylaxe zugelassenes AB-Präparat. Die Frage nach dem prophylaktischen Einsatz von AB beantworteten 110 der 111 Teilnehmer. Davon gaben 40% (44/110) an, AB prophylaktisch eingesetzt zu haben. Der prophylaktische Einsatz von AB erfolgte jeweils bei 31,8% (14/44) der Tierärzte bei 1–5% bzw. 6–25% ihrer Patienten.

Die häufigsten Indikationen, die zu einem prophylaktischen Einsatz führten, waren dabei eine drohende Sepsis beim Fohlen (24,3%, 27/44), grundsätzlich bei Operationen (20,7%, 23/44) und bei orthopädischen Operationen (13,5%, 15/44). In den vorangegangenen Experteninterviews wurde angeraten „orthopädische Operationen“ losgelöst von „bei Operationen grundsätzlich“ abzufragen.

108 der 111 Teilnehmer beantworteten die Frage nach dem Einsatz von AB für nicht antibakterielle Zwecke. 26,9% (29/108) gaben an, AB schon für nicht-antibakterielle Zwecke eingesetzt zu haben. Von diesen setzten 58,6% (17/29) AB bei weniger als 1% der Patienten und 31% (9/29) bei 1–5% der Patienten für nicht antibakterielle Zwecke ein. Eingesetzt wurden AB zur Sehnenerweichung (86,2%, 25/29), gegen Endotoxine (34,5%, 10/29), zur chemischen Ablation des Ziliarkörpers (17,2%, 5/29), zur Entzündungshemmung (13,8%, 4/29), zur Immunmodulation (10,4%, 3/29) und als Prokinetikum (3,5%, 1/29).

### Anwendung von nicht fixen AB-Kombinationen

**Abb. 5:**
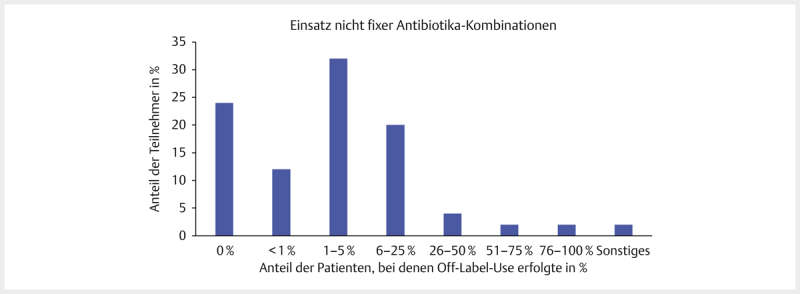
Einsatz von nicht fixen AB-Kombinationen nach Häufigkeit bezogen auf den Anteil der Patienten. Prozentuale Verteilung bezogen auf die Teilnehmerzahl, n=111. Quelle: M. Tarillion. **Fig. 5** Use of non-fixed antibiotic combinations by frequency. Percentage distribution in relation to the total number of participants, n=111. Source: M. Tarillion.

75,7% (84/111) der Teilnehmer gaben an, schon 2 und 27,4% (29/106) mehr als 2 verschiedenen AB zusammen eingesetzt zu haben, die nicht bereits vom Hersteller in einem Medikament enthalten waren. Von 82 Teilnehmern gaben 43,9% (36/82) an, AB-Kombinationen bei 1–5% ihrer Patienten einzusetzen und 26,8% (22/82) bei 6–25% ihrer Patienten. Die Häufigkeit ist in Abb. 5 nochmal in Bezug auf die Gesamtteilnehmerzahl von 111 Tierärzten dargestellt.

87 der 111 Teilnehmer machten Angaben zu den eingesetzten nicht fixen AB-Kombinationen. Die 3 meistgenannten Kombinationen, laut Umfrage, waren Gentamicin mit Penicillin (73,6%, 64/87), Gentamicin mit Amoxicillin (33,3%, 29/87) und Metronidazol mit Sulfonamid+TMP (21,8%, 19/87) ( Tab. 4 ).

**Table:** **Tab. 4** Die beliebtesten nicht fixen AB-Kombinationen in absoluter und relativer Häufigkeit bezogen auf die Teilnehmerzahl, n=87. Weitere gewählte und in Freitextkommentaren genannte Kombinationen: < 10: Gentamicin+Metronidazol, Gentamicin+Metronidazol+Penicillin, Amoxicillin+Penicillin, Amoxicillin+Clavulansäure+Gentamicin+Fluorchinolone, Sonstige: „Amoxi+Genta+ Metronidazol“, „Gentamicin Cephalosporin“, „Ofloxacin+Tobramycin“, „Chloramphenicol+Tobramycin“. **Table 4** The most popular non-fixed antibiotic combinations in absolute and relative frequency in relation to the number of participants, n=87. Other combinations selected and mentioned in free text comments: < 10: gentamicin+metronidazole, gentamicin+metronidazole+penicillin, amoxicillin+penicillin, amoxicillin+clavulanic acid+gentamicin+fluoroquinolones, other: “Amoxi+Genta+Metronidazole”, “Gentamicin+Cephalosporin”, “Ofloxacin+Tobramycin”, “Chloramphenicol+Tobramycin”.

Nicht fixe AB-Kombinationen	Häufigkeit absolut	Häufigkeit relativ (%) bezogen auf die Teilnehmerzahl, n=87
Gentamicin+Penicillin	64	74
Gentamicin+Amoxicillin	29	33
Metronidazol+Sulfonamid+TMP	19	22
Metronidazol+Amoxicillin	16	18
Tulathromycin+Rifampicin	15	17
Fluorchinolone+Penicillin	12	14
Azithromycin+Rifampicin	11	13

Der Einsatz erfolgte überwiegend bei fieberhaften/rezidivierenden Phlegmonen (54%, 47/87), bei Sepsis (51,7%, 45/87), bei akuten systemischen Infektionen zur Überbrückung der Wartezeit bis zum Antibiogrammergebnis (43,7%, 38/87) und bei Aspirationspneumonie und anderen Atemwegsinfekten (37,9%, 33/87). Gründe die zu dieser Anwendung geführt haben, waren v. a. die Notwendigkeit einer sofortigen Behandlung vor dem bakteriologischen Untersuchungsergebnis (77%, 67/87), die Problematik, dass kein TAM zugelassen ist (50,6%, 44/87) und Mischinfektionen (46%, 40/87).

### Therapieerfolg durch Off-Label-Use und Auswirkungen der neuen Rechtsprechung

Die Mehrheit der Teilnehmer gab an, dass sich durch den Off-Label-Use ein Therapieerfolg erzielen ließ ( Abb. 6 ). Ergänzend wurde die Frage gestellt, ob die Teilnehmer den Therapieerfolg durch die VO (EU) 2019/6 Artikel 106 Absatz 1 „TAM werden in Übereinstimmung mit Ihren Zulassungsbedingungen angewendet.“ als gefährdet ansehen. 72% sehen den Therapieerfolg bei strikter Einhaltung dieser Bestimmung als „gefährdet“ oder „stark gefährdet“ an (nicht abgebildet).

**Abb. 7:**
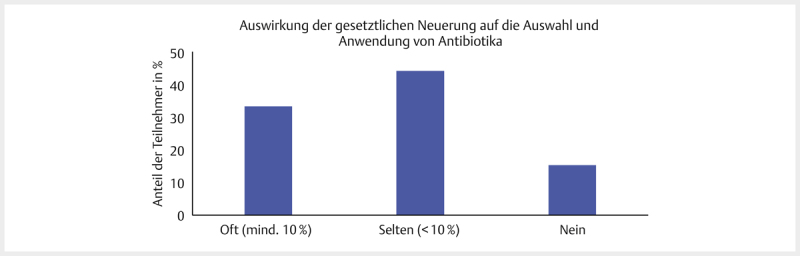
„Hat diese gesetzliche Neuerung Auswirkungen auf die Auswahl und Anwendung von antibiotischen Präparaten im Vergleich zu der Zeit vor der neuen Rechtslage/vor dem 28.01.2022?“ Relative Häufigkeit in Prozent bezogen auf die Teilnehmerzahl, n=111, SC-Frage. Quelle: M. Tarillion. **Fig. 7** “Does this legal novelty have an impact on the selection and use of antibiotic preparations compared to the time before the new legal situation/before 28.01.2022?” Relative frequency in percent in relation to the number of participants, n=111, SC question. Source: M. Tarillion.

Abb. 7 zeigt die Verteilung der Antworten auf die Frage, ob die neue Rechtslage Auswirkungen auf die Auswahl und Anwendungen von antibiotischen Präparaten hat. In einer separaten Frage sollten die Teilnehmer mit „Ja“ oder „Nein“ angeben, ob sich ihr Off-Label-Use durch die neue Rechtslage verändert hat. 55% (72/111) der befragten Tierärzte gaben an, dass sich seit der neuen Rechtslage nichts an ihrem Off-Label-Use geändert hat (nicht dargestellt). 33 der 111 Teilnehmer begründeten ihre Entscheidung in den Freitextkommentaren. Darunter waren die häufigsten Kommentare mit der Aussage, dass der Off-Label-Use insgesamt reduziert wurde (15/33) und dass Off-Label-Use z.T. unverzichtbar sei aufgrund von fehlenden Alternativen und dem Gefühl, dass Vorgaben in den Packungsbeilagen veraltet seien (8/33).

## Diskussion

Bei dieser Studie handelt es sich um die bisher umfassendste deutschlandweite Umfrage zum Thema Off-Label-Use in der Pferdemedizin. Durch diese Umfrage wurden erstmals Daten zum aktuellen Off-Label-Einsatz von AB bei Equiden in Deutschland nach dem Inkrafttreten der VO (EU) 2019/6 erhoben. Bezogen auf die Anzahl der vollständig beantworteten Rückmeldungen sind die Daten hinsichtlich der Repräsentanz limitiert, geben jedoch wichtige Einblicke in den Einsatz von AB in der Pferdepraxis. Demnach wenden die meisten Pferdepraktiker (88%) antibakteriell wirksame Arzneimittel abweichend von den Zulassungsbestimmungen an. Die hier präsentierten Informationen ermöglichen Rückschlüsse auf viele Aspekte des Off-Label-Uses, welche durch die zukünftige AB-Verbrauchsmengenerfassung bei Pferden nach Art. 57 VO 2019/6 nicht adressiert werden.

Bei dieser Umfrage lag zwar ein Schwerpunkt auf dem Off-Label-Use von den für Pferde zugelassenen AB, mit entsprechend genaueren Abfragen zum Umfang der Patienten, Art und Begründung, ergänzend wurden jedoch Fragen zur **Umwidmung** bezüglich Tierart bzw. Anwendungen von Humanarzneimitteln gestellt. Die Umwidmungen bezüglich Tierart durch viele Pferdepraktiker waren zu erwarten, weil es zum Zeitpunkt der Umfrageerstellung nur 10 (ein Wirkstoff davon doppelt abgefragt s. o.) für Pferde zugelassene antibakterielle Wirkstoffe in insgesamt 27 systemisch anzuwendenden Präparaten für ein schmales Spektrum der Anwendungsgebiete in Deutschland gab (VETIDATA, Stand: 13.07.2022). Hierüber wird das große Spektrum an bakteriellen Erkrankungen bei Equiden nicht abgedeckt. Auch die EMA kommt in einer 2017 veröffentlichten Übersicht 4 zu der Einschätzung, dass für viele Indikationen in der Pferdemedizin Therapienotstände wie beispielsweise bei Septikämien, *Rhodococcus-equi-* Infektionen, Anaplasmose, Borreliose und Clostridien-assoziierten Kolitiden bestehen.

Bei der **Umwidmung von TAM** standen laut unserer Umfrage Präparate mit Fluorchinolonen (Enrofloxacin, Marbofloxacin) und Breitspektrum-Cephalosporinen (Cefquinom, Ceftiofur) im Vordergrund, also Wirkstoffe aus der AMEG-Kategorie B 14 . Hierfür besteht bei Pferden, wie bei jeder Umwidmung eines AB nach Tierart, seit 2018 eine Antibiogrammpflicht 15 . In einer Umfrage von Pferdepraktikern in Großbritannien 16 , die sich auf bestimmte Fälle (z. B. Pyodermie) bezog, zeigten sich besonders in „Überweisungspraxen“ häufige Verschreibungen von Breitspektrum-Cephalosporinen (23%) und Enrofloxacin (10%), was die Bedeutung dieser als *critically important antibiotics* eingestuften „Reserve-AB“ 14 für schwer therapierbare Fälle nahelegt. Aus einer europaweiten Umfrage, die 25 EU-Länder einschloss, geht hervor, dass Fluorchinolone und Cephalosporine der 3. und 4. Generation je nach Indikation in bis zu 20% bzw. 22% der Fälle für Pferde verordnet wurden 17 . Die Beliebtheit von Fluorchinolonen wird auch durch deren orale Bioverfügbarkeit erklärt 18 . Grundsätzlich sollten Wirkstoffe aus der Kategorie B nach AMEG aber nur eingesetzt werden, wenn First-Line- (Kategorie D) und Second-Line-AB (Kategorie C) unwirksam sind 14 . Wie unsere Umfrage nahelegt, sollten diese Wirkstoffe und auch das durch viele Tierärzte umgewidmete Doxycyclin, als oral besser bioverfügbares Tetracyclin, in der bakteriologischen Diagnostik für Empfindlichkeitsbestimmungen in sogenannten Plattenlayouts 19 berücksichtigt werden. Metronidazol, das sich insbesondere zur Therapie von Anaerobier-Infektionen auszeichnet, darf nur bei Equiden, die von der Schlachtung ausgenommen sind, eingesetzt werden ( Tab. 2 der VO (EU) 37/2010). Der Aspekt des Equidenpass-Status blieb jedoch in der Umfrage unberücksichtigt. Zugunsten der Antwortrate wurde der Fragenumfang insgesamt begrenzt und arzneimittelrechtlich kompromittierende Fragen vermieden, so dass die Häufigkeit des Einsatzes bezogen auf Patienten, spezifische Gründe sowie die Durchführung von bakteriologischen Untersuchungen für umgewidmete TAM nicht abgefragt wurde. Spezifische Umfragen in Bezug auf Indikationen 16 17 bzw. auf die bakteriologische Diagnostik 20 sind hierzu auch besser geeignet. Zudem ist zu erwarten, dass sich der Umfang des Einsatzes dieser umgewidmeten AB aus den zukünftigen Meldungen der Verbrauchsmengen bei Equiden ergeben wird. Allerdings ergab sich aus der vom Zulassungsstatus unabhängigen Frage (also inklusive Umwidmung von TAM oder Humanarzneimitteln) zur Reihung der häufigsten Gründe für den Off-Label-Use nicht „Indikation“ in den ersten Positionen, wie wir aufgrund der mangelnden Verfügbarkeit von Pferdearzneimitteln erwartet hätten. Nach dem häufigsten Grund für den Off-Label-Use „Dosis“ wurde stattdessen „Packungsgröße“ genannt, was für einen Off-Label-Use prinzipiell unberechtigt ist. Insgesamt bestätigt diese Umfrage die gängige Praxis zur Umwidmung von TAM, die auf empirischen Daten in der Pferdemedizin basiert. Umwidmungen sind prinzipiell mit Unsicherheiten verbunden, zumal Literaturangaben zu Dosierungen i.d.R. wirkstoffbezogen sind und die pharmazeutischen Formulierungen eines Arzneimittels außer Acht lassen. Nur bei zugelassenen TAM wurde die Wirksamkeit und Verträglichkeit für die Zieltierart für die angegebenen Anwendungsgebiete nachgewiesen. So ist zum Beispiel die orale Gabe von Doxycyclin praktikabel, aber bei Pferden kann die orale Bioverfügbarkeit geringer sein als bei anderen Tierarten 21 .

Viele Pferdemediziner (51%) setzen laut dieser Umfrage zwar **Human-AB** ein, dies jedoch überwiegend bei einem kleinen Anteil ihrer Patienten. Wie sich aus der Frage zu nicht fixen Kombinationen ergab, fallen unter die Wirkstoffe Rifampicin und Azithromycin, die gegen *Rhodococcus-equi* -Infektionen in der sogenannten Positivliste als essenziell eingestuft wurden 22 . Da es diverse antibakterielle Wirkstoffe in Humantherapeutika gibt, wäre die Liste der Antwortoptionen zu lang gewesen. Um die Teilnehmer nicht zu überfordern, wurde auf eine genaue Abfrage dazu verzichtet. Die Anwendungsverbote, wie von Carbapenemen (z. B. Imipenem) gemäß der Durchführungsverordnung (EU) 2022/1255, führen nach Einschätzung der Mehrheit der Teilnehmer (77%) zwar nicht zu Therapielücken, aber rund ein Viertel sieht hierin Probleme, die weiter hinterfragt werden sollten. Die neue Durchführungsverordnung (EU) 2024/1973 (Commission Implementing Regulation [EU] 2024/1973 vom 18. Juli 2024) zu weiteren Beschränkungen zur Umwidmung einer Reihe von AB (u. a. Antibiogrammpflichten), war zum Zeitpunkt dieser Umfrage noch nicht in Kraft getreten und findet erst ab August 2026 Anwendung. Hierüber zeichnen sich für die Pferdemedizin jedoch ebenfalls keine wesentlichen Konsequenzen ab. Hinsichtlich solcher Folgeverordnungen sowie einer aktuellen Überarbeitung der „Positivliste für Equiden“ (VO [EU] 2019/6 Art. 115, Abs. 5) wären weiterführende Umfragen speziell zu Umwidmungen gemäß Art. 112 und Art 113 der VO (EU) 2019/6 sinnvoll.

Im Vordergrund dieser Umfrage stand der **Off-Label-Use von antibakteriell wirksamen TAM, die für Pferde zugelassen sind** . Die genaueren Abfragen können Hinweise auf sinnvolle Überprüfungen der Fachinformationen für ältere Produkte (Art 72 VO (EU) 2019/6) sowie für ein von der EMA angestrebtes Projekt zur Dosisrevision (ADRA) durch pharmazeutische Unternehmen liefern. Aus den Detailfragen zu einzelnen für Pferde zugelassenen AB konnten als Gründe überwiegend „Sofortige Behandlung vor bakteriologischem Untersuchungsergebnis notwendig“, „kein TAM für die Indikation zugelassen“, „Mischinfektionen“ und „keine ausreichende Wirkung aufgrund nicht optimaler Dosierung laut Zulassung“ identifiziert werden. Für Oxytetrazyclin ist der häufige Off-Label-Einsatz zur Sehnenerweichung hervorzuheben, was gängige Praxis in der Fohlenmedizin ist 23 . Aus Sicht der aktuellen tierarzneimittelrechtlichen Vorgaben und der Antibiotika-Leitlinien 6 , ist der nicht-antibiotische Einsatz von Oxytetrazyklin bei dieser Indikation nicht abgedeckt. Die in der Literatur beschriebenen Therapieempfehlungen 24 besitzen aufgrund des Fehlens aussagekräftiger klinischer Studien nur geringe Evidenz. Die in Freitextkommentaren erwähnte einmalige perioperative Anwendung von Oxytetrazyclin ist nicht konform mit der Leitlinie zur perioperativen Prophylaxe der British Equine Veterinary Association (BEVA) 25 , zumal es als bakteriostatisch wirksames AB eine zu kurze Eliminationshalbwertszeit hat 26 .

Bekanntlich werden die breit wirksamen potenzierten Sulfonamide in der Pferdepraxis sehr häufig eingesetzt 17 . Auffällig sind für die potenzierten Sulfonamide, wie für Präparate mit Sulfadimethoxin+TMP oder Sulfadiazin+TMP, die häufige Erhöhung der Dosis bis auf das Doppelte (40 mg/kg bzw. 60 mg/kg Gesamtwirkstoffmenge) bzw. eine zweimal tägliche Applikation der Tagesdosis. Ungeachtet der Zielspezies und des enthaltenen Sulfonamids ist in allen zugelassenen TAM ein Verhältnis der Sulfonamide zu TMP von 5:1 zu finden, obgleich sich die Pharmakokinetik, somit die zu erwartenden Gewebekonzentrationen, bei einzelnen Tierarten deutlich unterscheiden 27 . Daher erscheint fragwürdig, ob das angestrebte Konzentrationsverhältnis am Infektionsort (20:1) zur optimalen antibakteriellen Wirkung bei den üblichen Dosisvorgaben, die in der Regel nicht nach Tierarten differenzieren, erreicht werden 28 . Bei Pferden ist die TMP-Halbwertzeit mit ca. 3 Stunden sehr kurz 26 , wobei die Sulfonamid-HWZ je nach Wirkstoff variabel ist, sodass sich bei einmal täglicher Applikation eine TMP-freie Zeit mit subinhibitorischen Sulfonamid-Konzentrationen ergeben kann. Dies macht verständlich, dass Pferdepraktiker zu potenzierten Sulfonamiden manchen Empfehlungen aus der Fachliteratur zur Verkürzung der Dosisintervalle bzw. zur Dosiserhöhung bei einmal täglicher oraler oder parenteraler Verabreichung folgen 24 28 29 30 .

Auch die anderen für Pferde zugelassenen antibakteriellen Wirkstoffe wurden von vielen Tierärzten höher dosiert (vgl. Tab. 2 ), wobei dies nicht bestimmte Präparate betraf, somit offensichtlich nicht auf den Formulierungen der TAM basierte. Unterdosierungen sowie Verkürzungen der Behandlungsdauer, d. h. Faktoren, die bekanntlich Resistenzselektionen fördern können 28 , spielen hingegen laut dieser Umfrage keine Rolle in der Pferdemedizin. Abgesehen von der Tatsache, dass alle für Pferde zugelassenen AB seit Jahrzehnten, somit vor Inkrafttreten der strengen Zulassungsbestimmungen mit Wirksamkeitsprüfungen gegen bestimmte pathogene Bakterien, im Einsatz sind, fehlen für Pferde oftmals wichtige Indikationsangaben in Bezug auf Organsysteme/Erkrankungen in den Fachinformationen. So beschränkt sich das Anwendungsgebiet der 2 Injektionspräparate mit dem Aminoglykosid Gentamicin auf die Therapie von Infektionen der unteren Atemwege, weil die Wirksamkeit bei anderen Indikationen nicht klinisch geprüft wurde. Im Gegensatz zu anderen Tierarten fehlen wichtige Indikationen, wie Septikämie, wozu sich auch eine Kombination mit Penicillinen empfiehlt 28 . Daraus lässt sich die durch viele Tierärzte genannte Begründung „sofortige Behandlung“, „Indikation“ bzw. die Kombinationen (auch bei Mischinfektionen) ableiten. Trotz der nephro- und neurotoxischen Risiken von Gentamicin wenden einige Teilnehmer dieser Umfrage bei Einhaltung der Behandlungsdauer (3–5 Tage) Dosierungen von täglich 10–13,2 mg/kg i. v. statt 6,6 mg/kg i. v. an, was auch in der Literatur beschrieben ist 18 . Für dieses primär bakterizid wirksame Aminoglykosid sind die erreichten Spitzenkonzentrationen ausschlaggebend, so dass einmal tägliche Applikationen der Standarddosis von 6,6 mg/kg bei ambulant behandelten Pferden in der Regel ausreichen 31 . Auf Grundlage mancher pharmakokinetischer Studien finden sich jedoch Empfehlungen zu Dosierungen von ≥ 7,7 mg/kg bis 10 mg/kg i. v. täglich 32 . Der Nutzen vom Off-Label-Use bezüglich der Indikation bei Infektionen von Knochen und Gelenken, ist allerdings fragwürdig, weil Gentamicin keine gute Verteilung ins Knochengewebe aufweist 33 . Es bleibt unklar, ob die Teilnehmer Septikämien ausgehend von Knochen- oder Gelenksinfektionen bei dieser Angabe meinten, was spezifischere Umfragen in Bezug auf Indikationen bedürfte. Dies betrifft auch die häufig genannte Off-Label-Anwendung bei Abszessen, bei denen AB nur dann indiziert sind, wenn von einer bakteriellen Streuung auszugehen ist 13 .

Ein Off-Label-Use aufgrund „keine ausreichende Wirkung bei der Dosierung laut Zulassung” bestand für verschiedene zugelassene AB auch bezüglich einer Verlängerung der Behandlungsdauer (außer für Gentamicin). Hierbei ist allerdings zu beachten, dass es sich bei den Angaben zur Behandlungsdauer in den jeweiligen Fachinformationen oftmals um Empfehlungen handelt, wie z. B. für Präparate mit Benzylpenicillin-Procain „die Therapie sollte mindestens 3 Tage dauern und mindestens 2 Tage über das Abklingen der Symptome hinaus erfolgen”. In diesen Fällen ist eine längere Behandlung wie für 7 Tage nicht als Abweichung von der Fachinformation (den Zulassungsbedingungen) zu werten und somit kein Off-Label-Use wie kürzlich auch von der EMA bestätigt wurde 34 . Für Benzylpenicillin-Procain ist die Behandlungsdauer bei den für Pferde zugelassenen Präparaten zwar meist in dieser Weise mit 3–4 Tagen angegeben, kann aber auch 7 Tage betragen. Daher war bei der Umfrage auch stärker eine Verkürzung der Dauer von Interesse, was hinsichtlich Rezidiven und der Selektion von Resistenzen besonders kritisch ist 3 . Gemäß dieser Umfrage nahmen nur wenige Teilnehmer eine Verkürzung der Behandlungsdauer vor, die hierfür zum Beispiel Anwendungsschwierigkeiten oder intraartikuläre Applikationen als Gründe nannten.

Abgesehen von den potenzierten Sulfonamiden, sind keine antibakteriell wirksamen Kombinationspräparate für Pferde zugelassen. Entsprechend den Antibiotikaleitlinien ist der Einsatz von zugleich mehreren AB im Sinne von nicht fixen Kombinationen zwar wegen Resistenzselektionen möglichst zu vermeiden 6 , für bestimmte Anwendungsgebiete in der Pferdemedizin jedoch indiziert, so die Kombination aus Makroliden (Azithromycin oder Tulathromycin) plus Rifampicin gegen *Rhodococcus-equi* -Infektionen 4 . Aus dieser Umfrage geht hervor, dass weitere Kombinationen, die eine Erweiterung des Wirkungsspektrums mit sich bringen, in der Pferdepraxis aus überwiegend nachvollziehbaren Gründen eingesetzt werden, allem voran die Kombination aus Gentamicin mit Penicillinen, die zugleich synergistische Effekte hat 28 . Im Sinne einer Umwidmung gemäß Indikation wird diese Kombination laut unserer Umfrage neben einer drohenden Sepsis bei Fohlen auch oftmals zur perioperativen Prophylaxe angewendet, was den Ergebnissen einer US-amerikanischen Umfrage in der Pferdechirurgie entspricht 35 und insbesondere in der equinen Kolik-Chirurgie einen hohen Stellenwert hat 36 . Eine solche Breitspektrum-Antibiose zur Prophylaxe wird aber mit Risiken, wie einer hohen Ausscheidung von CTM-X produzierenden *E. coli* und nosokomialen postoperativen Infektionen, bei Pferden in Verbindung gebracht 35 37 . Die unter Einbeziehung von elektiven Operationen häufige (39–98%) perioperative Prophylaxe wird von der EMA hinsichtlich der geringen Inzidenz (0–0,9%) aller postoperativen Infektionen bei elektiven Eingriffen, wie Arthroskopie, kritisch gesehen 4 . In unserer Umfrage, die sich nicht speziell an chirurgisch tätige Pferdemediziner richtete, lag der Anteil der Tierärzte, die AB prophylaktisch bei Pferden einsetzen, bei 40%. Gemäß der VO (EU) 2019/6 (Art. 107, Abs. 3) ist eine antibakterielle Prophylaxe prinzipiell nur in Ausnahmefällen bei einzelnen Tieren erlaubt, wenn das Infektionsrisiko hoch und die Folgen wahrscheinlich schwerwiegend sind. Wünschenswert wären hierzu klarere Vorgaben, wie ein EU-Leitfaden für einen begründeten prophylaktischen Einsatz (perioperativ auch nach Art der Eingriffe) bei Pferden sowie Überprüfungen einzelner Empfehlungen 26 .

Wie die Umfrage zeigt, entsprachen die Therapieerfolge bei Off-Label-Use überwiegend den Erwartungen der Teilnehmer und der Großteil der befragten Tierärzte sieht dementsprechend den Erfolg der antibiotischen Therapie als gefährdet oder als stark gefährdet an, wenn sie sich immer strikt an die Zulassungsbedingungen halten müssten. Aus der Frage zur Auswirkung der neuen Rechtslage sowie Freitextkommentaren wurde deutlich, dass die befragten Tierärzte versuchen, den Off-Label-Use von AB weitestgehend einzuschränken, für sie aber das Wohl der Patienten an erster Stelle steht.

## Notes

Fazit für die Praxis
Wie diese Umfrage bestätigt, spielen Umwidmungen von Antibiotika (AB) in der Pferdemedizin eine große Rolle und besondere Bedingungen im Infektionsgeschehen rechtfertigen in vielen Fällen einen Off-Label-Use von zugelassenen Präparaten für Pferde, wie Dosiserhöhungen. Diese Möglichkeiten räumt das neue Tierarzneimittelrecht bei entsprechenden Begründungen, teils mit Vorgabe der Antibiogrammpflicht, auch durchaus ein. Die neue Rechtslage sollte in der Praxis jedoch zum Hinterfragen mancher Therapiekonzepte führen, was viele Teilnehmer dieser Umfrage durch Änderungen in der Verordnungspraxis von AB nach Inkrafttreten der VO (EU) 2019/6 erkennen lassen. Bei Überlegungen zu weiteren rechtlichen Reglementierungen zum AB-Einsatz, wie Durchführungsverordnungen, bleibt zu wünschen, dass die Anliegen, die aus Umfragen wie dieser ersichtlich werden, entsprechende Berücksichtigung finden. Alle für Pferde in Deutschland zugelassenen AB sind seit Jahrzehnten im Verkehr, so dass einige Angaben in der Fachinformation auf Erkenntnissen und Anforderungen von Zulassungsunterlagen basieren, die aus früherer Zeit stammen. Aus dieser Umfrage ergeben sich Hinweise darauf, dass manche Dosisangaben einer Überprüfung bedürfen. Harmonisierung der Fachinformationen und Dosisrevisionen sind ein erklärtes Ziel. Dies sollte jedoch nicht zum Verlust von den wenigen Pferdearzneimitteln führen, weil pharmazeutische Unternehmen möglicherweise die Kosten für klinische Prüfungen aufgrund fehlender finanzieller Anreize scheuen und ihre Produkte dann eher vom Markt nehmen. Empfehlungen zu Dosierungen von antibakteriellen Wirkstoffen, wie in der Fachliteratur beschrieben, können nicht die Besonderheiten von pharmazeutischen Formulierungen der verschiedenen Fertigarzneimittel abdecken. Kompromisse könnten dennoch in Leitfäden zur Antibiotikaauswahl sowie Dosierungen für bestimmte Indikationen bei Pferden bestehen, zu deren Erstellung Experten aus der Pferdepraxis und spezifische Umfragen beitragen können. Zudem ist das Pharmakovigilanzsystem ein sehr wichtiger Baustein zur Sicherheit von Arzneimitteln. Dazu können Pferdepraktiker durch Meldungen von unerwünschten Ereignissen (unerwünschte Wirkungen, fehlende Wirksamkeit) nach Anwendungen eines Antibiotikums gemäß Zulassungsbedingen oder nach einem Off-Label-Use einen ganz wesentlichen Beitrag liefern ( https://www.bvl.bund.de/VETUAW/DE/Home/home_node.html ).

## Zusätzliches Material

Zusätzliches Material Zusätzliches Material
