# Supplementary material for: Off-label use of antibiotics in equine medicine – an online survey in Germany
Source: Tierarztl Prax Ausg G Grosstiere Nutztiere. 2025 Jun 13;53(3):155–68. [Article in German] doi: 10.1055/a-2585-3269 (PMC12956439; doi:10.1055/a-2585-3269)
Supplement: Supplementary file 1 — Zusätzliches Material [file 10-1055-a-2585-3269-25853269-0001.pdf]

Zusätzliches Material

Zusatz-Material 1 Tabelle über die für Pferde zugelassenen antimikrobiellen Wirkstoffe und die zugelassenen Präparate zur systemischen Anwendung mit den Anwendungsvorgaben nach Fachinformation (VETIDATA, Stand 13.07.2022). i.m. = intramuskulär, i.t. = intratracheal, i.v. = intravenös, s.c. = subkutan, GK = Infektion von Gelenken und Knochen, GIT = Infektion des Gastrointestinaltrakts, HH = Infektion von Haut und Hautanhangsorganen (z.B. Klaue, Hufe, Pfote), RT = Infektion des Respirationstrakts, UGT = Infektion des Urogenitaltrakts (UGT), OT = Zur Behandlung von Infektionen, verursacht durch Oxytetracyklin-empfindliche Erreger, S = Septikämie, WA = Wunden und Abszesse.

Supplementary material 1 Table of antimicrobial active substances approved for horses and approved preparations for systemic use with the instructions for use according to the Summary of Product Characteristics (SPCs) (VETIDATA, as of 13.07.2022). i.m. = intramuscular, i.t. = intratracheal, i.v. = intravenous, s.c. = subcutaneous, GK = infection of joints and bones, GIT = infection of the gastrointestinal tract, HH = infection of skin and skin appendages (e.g., claw, hoof, paw), RT = infection of the respiratory tract, UGT = infection of the urogenital tract, OT = for the treatment of infections caused by oxytetracycline-susceptible pathogens, S = septicemia, WA = wounds and abscesses.

Wirkstoff	Präparat	Dosierung min (mg/kg)	Dosierung max (mg/kg)	Frequenz min	Frequenz max	Behandlungs-dauer min (in Tagen)	Behandlungs-dauer max (in Tagen)	Applikations art	Indikation
Amoxicillin	100 mg/ml	10	-	einmalig	-	einmalig	-	i.v., i.m., s.c.	RT, UGT, GIT, HH, S, WA
	200 mg/ml	10	-	2x tägl.	-	3	-	i.v., i.m., s.c.	RT, GIT
Ampicillin	100 mg/ml	10	-	3x tägl.	4x tägl.	3	5	i.v.	RT, GIT, UGT
Oxytetracyclin	100 mg/ml	3	10	1x tägl.	-	3	5	i.m., i.v.	OT
Benzylpenicillin-Procain	Nr. 1	21	-	1x tägl.	-	3	7	i.m.	RT, UGT, HH, GK
	Nr. 2	15	-	1x tägl.	-	3	-	i.m.	RT, UGT, HH, GK
	Nr. 3	15	-	1x tägl.	-	3	-	i.m.	RT, UGT, HH, GK
	Nr. 4	15	-	1x tägl.	-	3	-	i.m., s.c.	RT, UGT, HK, GK

	Nr. 5	15	-	1x tägl.	-	3	-	i.m., s.c.	RT, UGT, HK, GK, S
		20	-	2x im Abstand von 48 h	-	4	-	i.m., s.c.	RT, UGT, HH, GK, S
Sulfadimethoxin + TMP	Nr. 1	20	-	1x tägl.	-	3	7	oral	RT, GIT, UGT
	Nr. 2	20	-	1x tägl.	-	3	7	oral	RT, GIT, UGT
	Nr. 3	20	-	1x tägl.	-	3	7	oral	RT, GIT, UGT
Sulfadoxin + TMP	Nr. 1	15	-	1x tägl.	-	3	5	im., i.v., i.t.	RT, UGT, HH, GK
	Nr. 2	15	-	1x tägl.	-	3	5	i.v.	RT, GIT, UGT, GK
Sulfadiazin + TMP	Nr. 1	15	-	2x tägl.	-	3	5	oral	RT, UGT, GIT, HH, GK
	Nr. 2	30	-	1x tägl.	2x tägl.	5	-	oral	RT, UGT
	Nr. 3	5 mg Trimethoprim + 25 mg Sulfadiazin pro kg KGW		1x tägl.	-	5	-	oral	RT, UGT, GIT, HH
	Nr. 4	15	-	2x tägl.	-	3	5	oral	RT, UGT, GIT, HH, GK
	Nr. 5	15	-	2x tägl.	-	3	5	oral	RT, UGT, GIT, HH, GK
Sulfadimidin	Nr. 1	50	65	1x tägl.	-	2	6	oral	RT, UGT, GIT, HH, GK
	Nr. 2	50	100	1x tägl.	-	3	7	oral	RT, UGT, GIT, HH

 © 2025. Thieme. All rights reserved.

	Nr. 3	50	-	1x tägl.	-	5	7	i.v., i.m., s.c.	RT, UGT, S
Sulfadimidin + TMP	Nr. 1	16	24	1x tägl.	-	3	5	i.v.	RT, UGT, GIT, HH
	Nr. 2	16	24	1x tägl.	-	3	5	i.v.	RT, UGT, GIT, HH
	Nr. 3	16	24	1x tägl.	-	3	5	i.v.	RT, UGT, GIT, HH
Gentamicin	Nr. 1	6,6	-	1x tägl.	-	3	5	i.v.	RT
	Nr. 2	6,6	-	1x tägl.	-	3	5	i.v.	RT

Zusatz-Material 2 Fragebogen zum Thema „Off-Label-Use von Antibiotika in der Pferdemedizin“ für in Deutschland praktizierende Tierärzte mit dem Behandlungsschwerpunkt Pferde. Quelle: M. Tarillion

Supplementary material 2 Questionnaire on “Off-label use of antibiotics in equine medicine” for veterinarians practicing in Germany with a focus on horses. Source: M. Tarillion

Final-Umfrage Pferde – Fragenübersicht

Demographische Fragen

1. Sind Sie angestellter oder selbstständiger Tierarzt?
2. Bei welcher Tierart liegt Ihr Behandlungsschwerpunkt?
3. Welcher Fachrichtung fühlen Sie sich am ehesten zugehörig?
4. Welche Qualifikationen haben Sie inne?
5. Seit wann besitzen Sie Ihre Approbation?
6. In welcher Praxisstruktur arbeiten Sie?
7. Wo ist die Praxis/Klink gelegen?
8. In welchem Bundesland ist die Praxis/Klinik gelegen?

Allgemeine Fragen

1. Haben Sie Antibiotika in der Vergangenheit (in den letzten zwei Jahren) off-label eingesetzt?
    a. Wie häufig haben Sie AB off-label eingesetzt?
2. Welche für Pferde zugelassenen Antibiotika haben Sie in der Vergangenheit (in den letzten zwei Jahren) am häufigsten off-label angewandt?
3. Welche tiermedizinisch zugelassenen AB, die nicht für Pferde zugelassen sind, haben Sie in den letzten zwei Jahren angewandt?
4. Bei welcher infektionsbedingten Indikation haben Sie AB off-label angewandt?
5. Was waren die häufigsten Gründe für einen Off-Label-Use von AB?
6. Woran orientieren Sie sich bei der Dosierung von AB?
7. Hat sich seit der neuen Rechtslage, die seit dem 28.01.2022 gilt (s. unten), etwas an Ihrem Off-Label-Use geändert und wenn ja, wie?
8. Wird das Gewicht der Patienten vor der Behandlung bestimmt?
    1. Wie erfolgt die Gewichtsbestimmung?
    2. Wann erfolgt die Gewichtsbestimmung bei der Behandlung mit AB?

Prophylaxe

1. Haben Sie AB prophylaktisch eingesetzt?
    a. Wie häufig haben Sie AB prophylaktisch eingesetzt?
    b. Bei welcher Indikation haben Sie AB prophylaktisch eingesetzt?

Einsatz für nicht-antibakterielle Zwecke

1. Haben Sie AB für nicht-antibakterielle Zwecke eingesetzt?
    a. Wie häufig haben Sie AB für nicht-antibakterielle Zwecke eingesetzt?
    b. Bei welcher Indikation haben Sie AB für nicht-antibakterielle Zwecke eingesetzt?
    c. Aus welchem Grund haben Sie sich für diesen Off-Label-Use von AB entschieden?

Nicht als Fertigarzneimittel zugelassene Antibiotika-Kombinationen

1. Haben Sie schon zwei verschiedene tiermedizinische AB zusammen eingesetzt, die nicht bereits vom Hersteller in einem Medikament enthalten waren?

2. Haben Sie schon mehr als zwei tiermedizinische AB zusammen eingesetzt, die nicht bereits vom Hersteller in einem Medikament enthalten waren?
    a. Wie häufig haben Sie zwei oder mehr tiermedizinische AB zusammen eingesetzt, die nicht bereits vom Hersteller in einem Medikament enthalten waren?
    b. Welche tiermedizinischen AB-Kombinationen haben Sie eingesetzt, die nicht bereits vom Hersteller in einem Medikament enthalten waren?

8

    c. Für welche Indikation haben Sie tiermedizinische AB-Kombinationen aus zwei oder mehreren AB eingesetzt, die nicht bereits vom Hersteller in einem Medikament enthalten sind?
    d. Aus welchem Grund haben Sie sich für den Off-Label-Use von AB entschieden?

Humanmedizinische Antibiotika

1. Haben Sie Antibiotika-Präparate aus der Humanmedizin umgewidmet?
2. Wie häufig haben Sie Antibiotika aus der Humanmedizin umgewidmet?
3. Aus welchem Grund haben Sie Antibiotika aus der Humanmedizin umgewidmet, deren Wirkstoffe auch in tiermedizinischen Produkten enthalten sind?
4. Bedeutet ein Einsatzverbot folgender Gruppen für die Tiermedizin ein therapeutisches Problem für Ihre tierärztliche Arbeit?

Therapieerfolg

1. Wie häufig entsprach der Therapieerfolg durch den Off-Label-Use von Antibiotika Ihren Erwartungen?
2. Sehen Sie den therapeutischen Erfolg der antibiotischen Behandlung durch die Regel von Artikel 106 Absatz 1 "Tierarzneimittel werden in Übereinstimmung mit den Zulassungsbedingungen angewendet." als gefährdet an?
3. Hat diese gesetzliche Neuerung Auswirkungen auf die Auswahl und Anwendung von antibiotischen Präparaten im Vergleich zu der Zeit vor der neuen Rechtslage/vor dem
9 28.01.2022?

Dosierungsfragen

Schema: Sie haben angegeben **xy** (Präparate, z.B. ...) off-label verwendet zu haben. Wie und wofür wurde es **hauptsächlich** off-label angewandt?

- **Indikation**: Infektion bzw. Infektion von...
- **Dosis** bezogen auf die Gesamtwirkstoffmenge je Einzelgabe:
- **Frequenz**:
- **Applikationsart**:
- **Behandlungsdauer**:
- Aus welchem **Grund** haben Sie sich für den Off-Label-Use von **xy** entscheiden?

10

11
